# Impact of the COVID-19 pandemic on inpatient antibiotic use in Indonesia and the Philippines

**DOI:** 10.1017/ash.2023.209

**Published:** 2023-09-29

**Authors:** Amara Fazal, Olivia McGovern, Garrett Mahon, Fernanda Lessa, Ma Tarcela Gler, Jemelyn Garcia, Mark Festin, Kuntaman Kuntaman, Ida Parwati, Cherry Siregar, Jay Christian, Gina de Guzman Betito, Maya Montemayor, Arleen De Leon, Emmeline Borillo, Mark Llanes, Regina Berba, Musofa Rusli, Farizal Rizky, Mariyatul Qibtiyah, Bambang Semedi, Rosantia Sarassari, Leonardus Widyatmoko, Basti Andriyoko, Adhi Sugianli, Dewi Turbawaty, Ivo Ranita, Franciscus Ginting, Made Krisna, Rahmadania Marita Joesoef, Twisha Patel

## Abstract

**Background:** The coronavirus disease 2019 (COVID-19) pandemic severely affected Southeast Asia, with >35 million cases and ~360,000 deaths. Despite relatively low rates of secondary bacterial infection among inpatients with COVID-19, several countries reported increased antibiotic use; raising concerns for worsening antimicrobial resistance. We assessed the impact of the COVID-19 pandemic on the use of antibiotics commonly used to treat respiratory infections in Southeast Asia. **Methods:** We evaluated intravenous antibiotic use among hospitalized adults in acute-care wards in 6 hospitals; 3 in Indonesia and 3 in the Philippines. We abstracted data on antibiotics that are commonly used to treat respiratory infections in these hospitals. We calculated antibiotic use rates for the 25 included antibiotics as monthly defined daily dose per 1,000 patient days (or patient discharges where patient days was unavailable) using data from pharmacy dispensing records and administrative records. Median antibiotic use rates for the prepandemic period (March 2018–February 2020) and the pandemic period (March 2020–February 2021) were compared, and percentage changes were calculated for (1) all 25 antibiotics combined; (2) ceftriaxone; (3) vancomycin and linezolid combined (anti-MRSA); and (4) broad-spectrum antibiotics with activity against *Pseudomonas aeruginosa* (anti-PSA). Monthly antibiotic use and COVID-19 patient discharges were graphed over the 36-month study period (March 2018–February 2021) to visualize trends (Fig. 1). The Wilcoxon rank-sum test was used to determine whether differences in median antibiotic use rates were statistically significant (2-tailed *P* < .05). **Results:** Overall, trends in antibiotic use were higher during months with increased COVID-19 patient discharges (Fig. 1). Use of all 25 antibiotics combined significantly increased in 4 of 6 hospitals (6.9%–63.6%) during the pandemic period compared to the prepandemic period. Ceftriaxone use significantly increased in 3 of 6 hospitals (37.1%–55.4%) and decreased in 3 of 6 hospitals (15.9%–31.9%). Anti-PSA antibiotic use significantly increased in 4 of 6 hospitals (16.1%–161.5%). Although anti-MRSA antibiotic use was low (comprising <2% of the overall included antibiotic use in Indonesia and <11% in the Philippines), use during the pandemic increased in 3 of 6 hospitals (59.8%–212.6%). **Conclusions:** We observed substantial increases in antibiotic use among hospitalized adults in Indonesia and the Philippines during the COVID-19 pandemic. The increased use of broad-spectrum antibiotics is concerning given the potential consequence of worsening antimicrobial resistance. Understanding how increases in antibiotic use compares to rates of bacterial infection, antimicrobial resistance, and antibiotic availability and accessibility during this time is important to contextualize results. These findings reinforce the importance of antibiotic stewardship practices to optimize antibiotic use, especially during pandemics.

**Figure:**
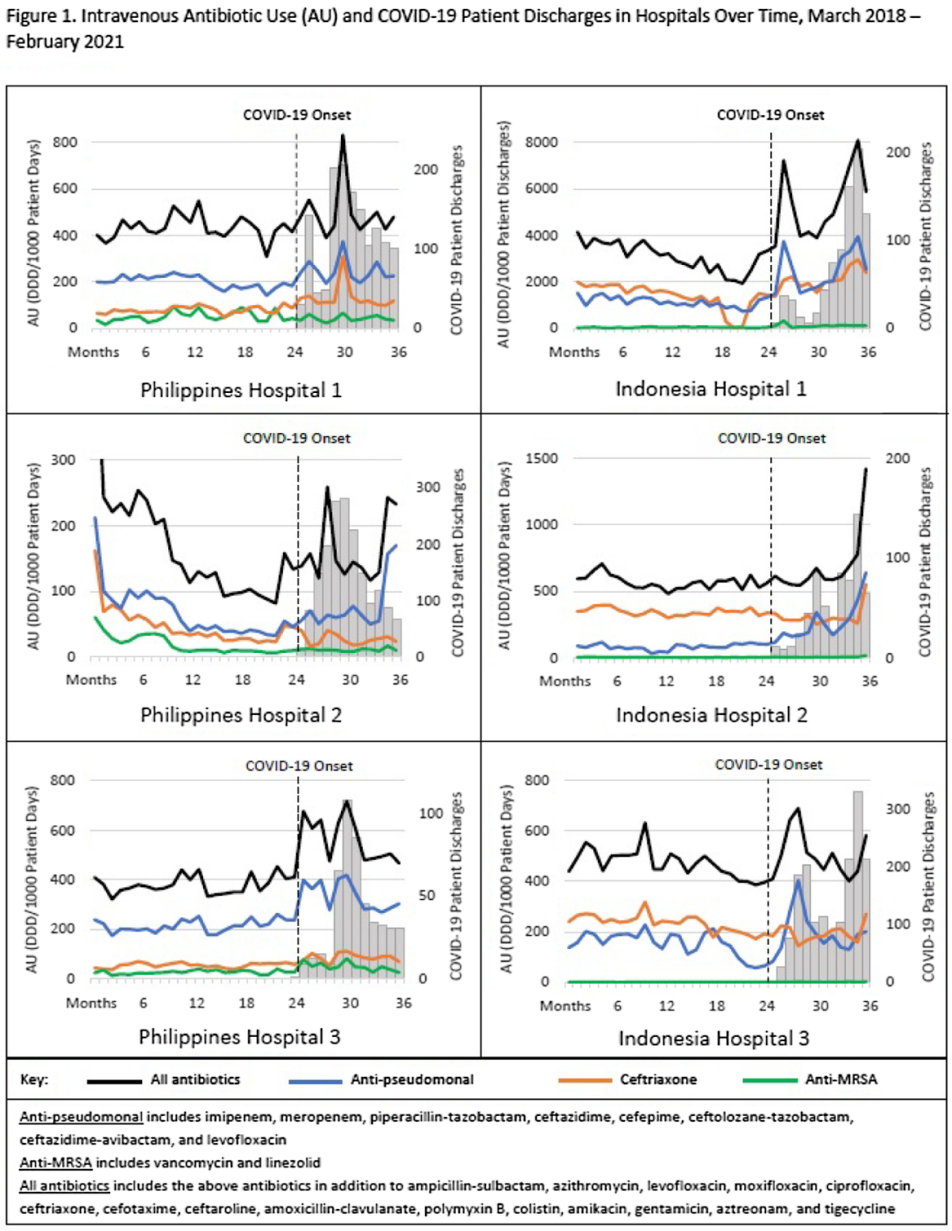

**Disclosure:** None

